# Triphala in Traditional Ayurvedic Medicine Inhibits Dengue Virus Infection in Huh7 Hepatoma Cells

**DOI:** 10.3390/ph14121236

**Published:** 2021-11-28

**Authors:** Aussara Panya, Kanyaluck Jantakee, Suthida Punwong, Supawadee Thongyim, Thida Kaewkod, Pa-thai Yenchitsomanus, Yingmanee Tragoolpua, Hataichanok Pandith

**Affiliations:** 1 Department of Biology, Faculty of Science, Chiang Mai University, Chiang Mai 50200, Thailand; kanyaluckjan@gmail.com (K.J.); tda007suju@gmail.com (T.K.); yboony150@gmail.com (Y.T.); 2 Research Center in Bioresources for Agriculture, Industry and Medicine, Faculty of Science, Chiang Mai University, Chiang Mai 50200, Thailand; 3 Doctoral Program in Applied Microbiology (International Program), Faculty of Science, Chiang Mai University, Chiang Mai 50200, Thailand; mildmsp@gmail.com; 4 Doctoral Program in Biology, Faculty of Science, Chiang Mai University, Chiang Mai 50200, Thailand; supawadeethongyim@gmail.com; 5 Division of Molecular Medicine, Research Department, Faculty of Medicine Siriraj Hospital, Mahidol University, Bangkok 10700, Thailand; ptyench@gmail.com

**Keywords:** Triphala, *Terminalia bellirica* extract, gallic acid, anti-dengue virus activity, cytokine storm

## Abstract

Traditional Triphala (three fruits), consisting of *Phyllanthus emblica, Terminalia chebula,* and *Terminalia bellirica,* presents a broad range of biological activities. However, its ability to inhibit dengue virus (DENV) infection has not been reported yet. Herein, the authors investigated the efficiency of three different Triphala formulations and its individual extract constituents to inhibit DENV infection. Treatment with *T. bellirica* extract or Triphala formulated with a high ratio of *T. bellirica* extract showed remarkable efficiency in significantly lowering DENV infection in Vero cells. Their effects were further studied in Huh7 cells, to address its potential ability in human cells. Treatment with 100 μg/mL of *T. bellirica* extract or Triphala resulted in an approximate 3000-fold or 1000-fold lowering of virus production, respectively. Furthermore, the treatment diminished *IL-6* and *CXCL-10* expressions, which are the hallmark of the cytokine storm phenomenon in DENV infection. The HPLC profiling demonstrated gallic acid as a major compound, the treatment by which showed its ability to effectively inhibit DENV infection after virus entry. Molecular docking demonstrated that gallic acid was able to interact with DENV NS5 protein, which could be one of Triphala’s antiviral mechanism. This study offers Triphala formulation and its ingredient, *T. bellirica* extract, as a natural based pharmaceutical to be used in DENV infection treatment.

## 1. Introduction

Natural products serve as valuable sources of bioactive substances which have been used in medical purpose since ancient times. Based on traditional uses, the natural substances and their metabolites provided either therapeutic potential or a great degree of safety to be applied in modern drug discovery and pharmaceuticals. Triphala (three fruits) is one of the most well-known herbal formulation in ayurvedic medicine and its therapeutic potential has been reported for a wide range of diseases. The Triphala formulation is a combination of *Phyllanthus emblica, Terminalia chebula,* and *Terminalia bellirica,* the biological properties of which are well documented in relation to the treatment of cancer, immunomodulation and antioxidants, as are its antimicrobial properties [1]. In addition to the diverse uses of Triphala, the antiviral activity of *T. chebula* has been reported to inhibit hepatitis B viral infection [2]. Treatment with *T. chebula* extract was capable of inhibiting hepatitis B virus multiplication by lowering viral DNA and surface antigen (HBsAg) levels in the extracellular medium of HepG2 2.2.15 cells [2]. The mechanism was explored and its inhibitory activity was revealed via the inhibition of hepatitis B virus DNA polymerase, and the modulation of TH1 and TH2 cytokine secretions [3], indicating the benefits of Triphala in treating infectious diseases. Unfortunately, so far only a few studies have reported Triphala antivirus activity by directily inhibiting virus infection or replication. Certainly, no anti-viral activity of Triphala against flaviviruses (such as hepatitis C, West Nile virus, Zika virus, and dengue virus (DENV) has been reported.

DENV belongs to the *Flaviviridae* family [4]. The infectious virion is coated with envelope proteins to seal the nucleocapsid inside. The genetic material is positively single-stranded RNA of approximately 11 kilobases in length. The genomic RNA, capping with m7GpppAmp at 5′ but lacking a poly A tail at 3′, encodes for 10 proteins of the virus including structural proteins (capsid, prM, envelope) and nonstructural proteins (NS1, NS2A, NS2B, NS3, NS4A, NS4B and NS5) [4]. Four closely related serotypes of DENV (DENV1, DENV2, DENV3 and DENV4) circulate and are transmitted from infected human to human via the bite of *Aedes aegypti* or *Aedes albopictus* mosquitoes [4]. The infection of DENV can cause a spectrum of diseases ranging from mild dengue fever (DF) to more severe diseases such as dengue hemorrhagic fever (DHF) and dengue shock syndrome (DSS) which can lead to the death of the infected patients [5]. DENV infection emerges as a critical public health problem in tropical and subtropical areas where four serotypes of DENV are commonly co-circulating. Over the past five decades, DENV infection spread throughout several geographic regions around the world [6]. DENV infection causes a serious impact not only on the quality of life but also long-term impacts on the economy. A dramatic increase of incidences [7] means more than 2.5 billion people (40% of the world’s population) are currently at risk from DENV infection [7]. Annually, the World Health Organization (WHO) estimates 50–100 million DENV infections worldwide, approximately 500,000 of which become severe cases (mostly children) and require hospitalization [7]. The mortality rate is estimated at 2.5% of those suffering from severe DENV infection. There is still no effective therapeutic option for DENV infection treatment. The effectiveness of the licensed vaccine Dengvaxia^®^ (CYD-TDV), developed by Sanofi Pasteur, was varied to protect the serotypes of DENV. It has been reported as a suboptimal protection against DENV1 and DENV2 with 50% and 35–42% protection, respectively [8,9,10]. The WHO recommends Dengvaxia^®^ for the endemic countries where the infection occurs regularly throughout the year [7]. Along with vaccination, which ultimately aims to lower the incidence of DENV infection, the development of a therapeutic agent is still needed to minimize the risk of severity and control the spread of the disease. Although there have been several attempts to develop an effective drug against DENV, so far, no anti-viral agent has been approved for DENV treatment.

To demonstrate the potential of Triphala in inhibiting DENV infection, therefore, the present study investigated the antivirus activity of Triphala and its extract constituents in inhibiting DENV infection in vitro. The efficiency of different Triphala formulations and extract compositions was compared in Vero cells. The most effective extracts and formulations were further studied for their effects in lowering the infected cell number, the intracellular viral protein levels, virus production and viral genomic material in hepatocellular Huh7 cells which mimics the natural target of DENV to emphasize its potential in human treatment.

## 2. Results

### 2.1. The Antiviral Effects of Triphala and Its Individual Constituents against DENV Infection in Vero Cells

*P. emblica, T. chebula,* and *T. bellirica* which are the constituents of Triphala was evaluated for the antiviral activity against DENV-2 infection, which is the most virulent serotype in Vero cells. Triphala was prepared in three different formulations, according to the traditional Thai medicine, so-called “Maha Pikud Triphala” in which all formulations contained *P. emblica, T. chebula,* and *T. bellirica* extracts but at different ratios. Three formulations, F1, F2 and F3, in addition to the individual extracts were firstly evaluated for their toxicity to the cells using a cell viability assay (Figure S1, Supplementary Materials). The non-toxic concentrations (12.5–100 μg/mL) were selected to investigate the effects in lowering the levels of intracellular viral proteins, using ELISA.

**Figure 1:**
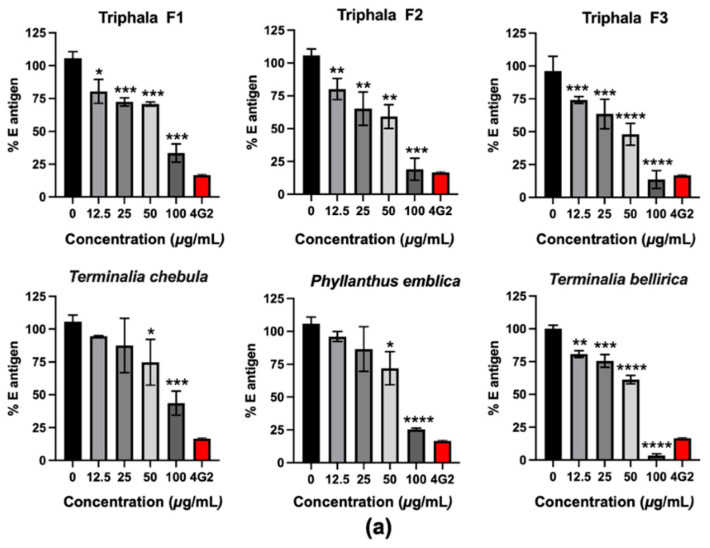

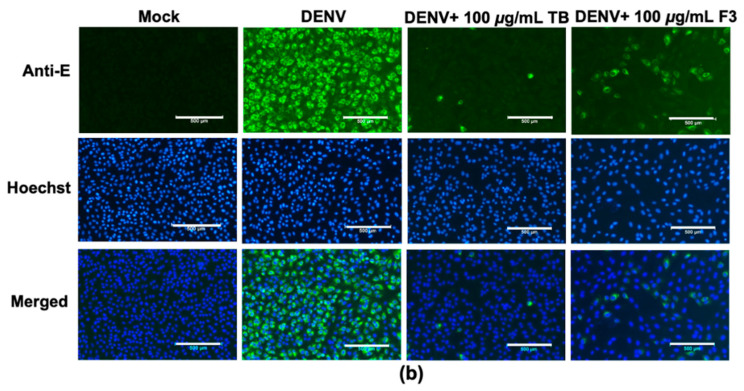
Antiviral activity of Triphala formulations and extracts against DENV-2 infection in Vero cells. (**a**) The antiviral activity of three formulations of Triphala and their extract components including *P. emblica*, *T. chebula*, and *T. bellirica* were found to inhibit viral infection by using cell-based ELISA to detect intracellular viral E antigen relative to that of non-treatment control. The neutralizing monoclonal antibody called 4G2 was used as the positive control to inhibit DENV infection (red bar). The statistical difference was tested by using Student’s *t*-test which * indicates *p* < 0.05; ** indicates *p* < 0.01; *** indicates *p* < 0.001 and **** indicates *p* < 0.0001. (**b**) Antiviral activity of *T. bellirica* (TB) extract and Triphala formulation 3 (F3) in decreasing virus infectivity in Vero cells was measured. The DENV-2 infection cells were treated with 100 μg/mL of TB extract or F3 and measured for the level of virus infectivity using IFA at 48 h post of infection.

Treatment with Triphala formulations or the individual extracts therefrom significantly decreased the percentage of E antigen, relative to non-treatment control in a dose-dependent manner (Figure 1a). At the highest test concentration (100 μg/mL), *T. bellirica* extract and Triphala F3 (*P. emblica, T. chebula,* and *T. bellirica* ratio of 3:1:2) significantly reduced the percentage of E antigen to 3.51% and 13.56%, respectively. The current research further confirmed the inhibitory effect of *T. bellirica* extract and Triphala F3 on the reduction in the number of the infected cells accessed by immunofluorescence assay (IFA). The number of infected cells (in green) in the presence of 100 μg/mL of *T. bellirica* extract or Triphala F3 was considerably lower than that of the non-treated control (Figure 1b). The results reflected the efficiency of the *T. bellirica* extract and Triphala F3 formulation in inhibiting DENV infection.

### 2.2. Gallic Acid as a Major Bioactive Compound in P. emblica, T. chebula, and T. bellirica Extracts

**Figure 2:**
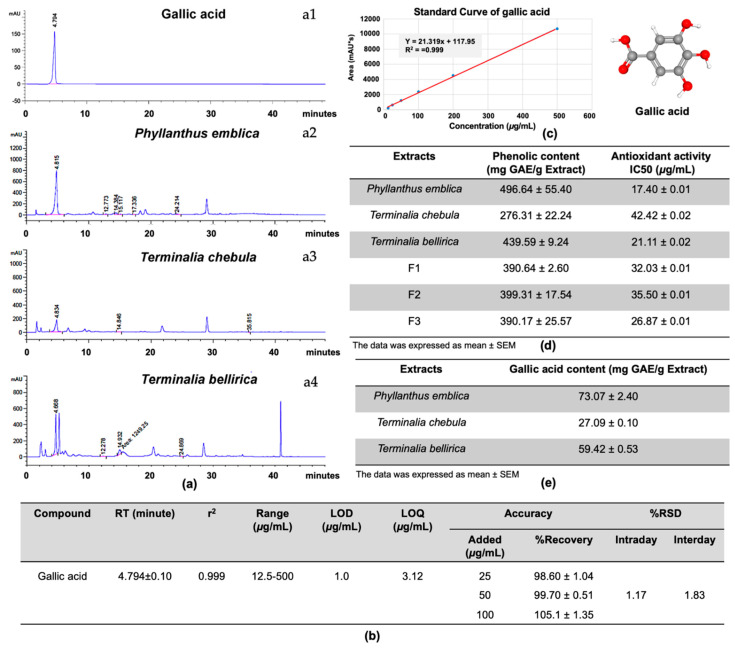
HPLC profile. The Triphala extracts were analyzed by using HPLC and compared to the standard gallic acid at RT4.794 (**a1**), *P. emblica* at RT 4.815 (**a2**), *T. chebula* at 4.834 (**a3**), and *T. bellirica* at 4.668 (**a4**). The HPLC method was validated by defining the linearity, accuracy and precision, LOD and LOQ (**b**,**c**). All the validation factors were calculated by n = 3. The total phenolic content and antioxidative activity of each plant were evaluated by using Folin–Ciocalteu method and DPPH free radical method, respectively (**d**). Gallic acid content in the individual extract was analyzed relative to the standard gallic acid by using HPLC and showed as mean ± standard error of mean (**e**).

The bioactive compounds of *P. emblica, T. chebula, and T. bellirica* were confirmed, according to data from previous literature that suggested gallic acid as the major active compound in the extracts. HPLC was performed and clearly demonstrated gallic acid as the main compound of the extracts (Figure 2a). The HPLC method was validated by defining the linearity, accuracy, and precision, LOD and LOQ (Figure 2b,c). A high repeatability in the retention time was obtained. The standard curve of gallic acid showed linearity at the concentration of 12.5–500 μg/mL. The regression equation was presented as y = 21.319x + 117.95. Values of the regression coefficients (R^2^) of the markers were higher than 0.999 (Figure 2c). LOD and LOQ values were 1.0 μg/mL and 3.12 μg/mL, respectively (Figure 2b). The high recovery values (98.60–105.1%) indicated the high repeatability of the proposed method (Figure 2b). The relative standard deviation of all parameters, inter-day, was 1.17% and, intra-day, was 1.83%, which, being less than 2.0%, revealed that the proposed method was precise (Figure 2b). Therefore, this HPLC method can be regarded as selective, accurate and precise for the quantification of gallic acid in Triphala.

The total phenolic compounds varied among the extracts and Triphala formulations, ranging from 276.31 ± 22.24 to 496.64 ± 55.40 mg of GAE (Figure 2d), whereas no remarkable differences were found among the three formulations of Triphala, which contained approximately 390.17 ± 25.27 to 399.31 ± 17.54 mg of GAE. The observed total phenolic contents corresponded to the amounts of gallic acid calculated from areas under the HPLC peak (Figure 2e). The *P. emblica* contained the highest amount of gallic acid with 73.07 ± 2.40 mg of gallic acid; *T. bellirica* extract contained the second highest amount, 59.42 ± 0.53 mg. The *T. chebula* extract had the lowest amount of gallic acid at 27.09 ± 0.10 mg. These amounts of gallic acid and total phenolic acid were coordinated with their antioxidative activity. The higher amount of *T. bellirica* and *P. emblica* in formulations F3 and F1, respectively, also exhibited higher antioxidative activity than F2, although F2 contained higher amounts of total phenolic compounds. These results suggested that gallic acid in *T. bellirica* and *P. emblica* might be the most effective bioactive components among other phenolic compounds (Figure 2d).

### 2.3. Gallic Acid Inhibited DENV Infection in Different Stages

**Figure 3:**
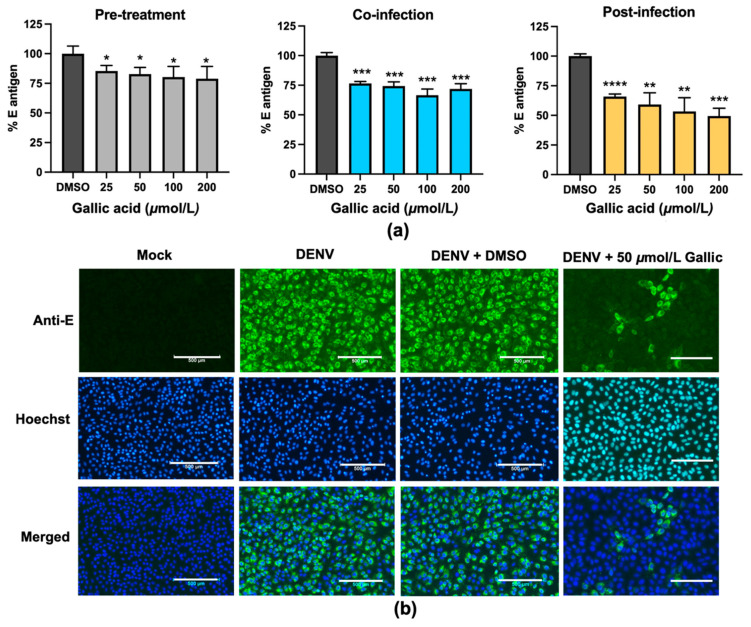
Antiviral activity of gallic acid to inhibit DENV infection in Vero cells. Various concentrations (25–200 μmol/L) of gallic acid were tested for antiviral activity against DENV-2 at different treatment conditions, including (**a**) pre-treatment, co-infection, and post-infection using cell-based ELISA assay to detect intracellular viral envelope (E) antigen. The statistical difference was tested by using Student’s *t*-test which * indicates *p* < 0.05; ** indicates *p* < 0.01; *** indicates *p* < 0.001 and **** indicates *p* < 0.0001. (**b**) the most effective activity of gallic acid in the post-infection condition was confirmed to reduce viral infectivity by using IFA.

To study the stage of gallic acid action in inhibiting DENV infection, we varied the addition time of when several concentrations of gallic acid (25–200 μmol/L) were added to the virus before infection (pre-treatment), during virus infection (co-infection), and after the virus entered the cells (post-infection). The results demonstrated that gallic acid treatment could reduce the percentage of E antigen relative to the non-treatment control in a dose-dependent manner in all tested conditions. At the highest tested concentration (200 μmol/L), gallic acid decreased the E antigen to 78.86, 71.96, and 49.38%, relative to the nontreatment control in pre-treatment, co-infection, and post-infection, respectively (Figure 3a). The most effective treatment was observed in the post-infection condition. To emphasize the inhibitory effect of gallic acid, the immunofluorescence staining (IFA) was conducted to determine the effect on the reduction of infected cell numbers. The treatment of gallic acid at the post-infection stage had a dramatic effect in lowering the number of infected cells compared with the nontreatment control (Figure 3b).

**Figure 4:**
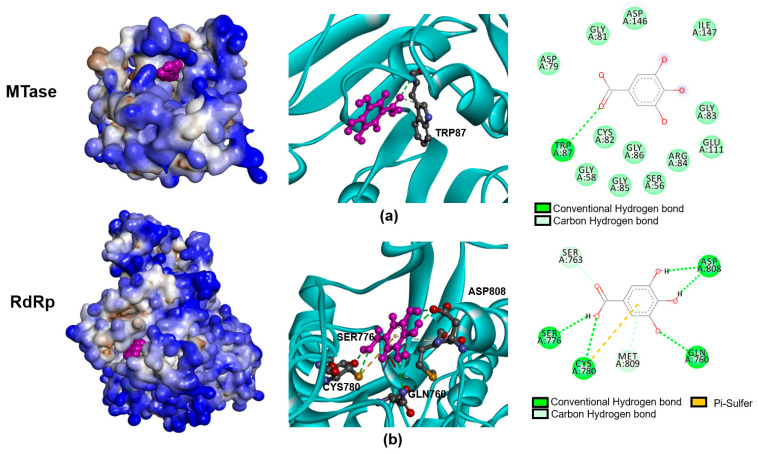
The interactions of gallic acid and DENV NS5 proteins. The simulation of ligand–protein interactions between gallic acid and NS5 MTase (**a**) and NS5 RdRp (**b**) was conducted using Swissdock (http://www.swissdock.ch/ accessed on 19 November 2021). The docking poses were analyzed for the interaction of gallic acid (magenta) and the residues of the protein targets (ball and stick) by using Discovery Studio software (**left** and **middle**). The detailed interaction and key binding residues were demonstrated as 2d diagrams to indicate the bonds linked between ligand and receptor (**right**).

The most effective activity of gallic acid was observed in the post-infection condition, which suggested that the gallic acid might affect the infection at the post-entry step (after the virus has entered to the host cells). The interaction of gallic acid and viral proteins possibly contributed to this biological function; therefore, molecular docking was performed to illustrate the interaction of gallic acid with the NS5 protein, which plays a major role in viral RNA replication. Interestingly, the docking simulation showed gallic acid could bind to NS5 MTase (PDB entry 1R6A), and NS5-RdRp (PDB entry 5JJR) with docking scores of −7.49, and −7.35. The analysis of intermolecular interaction showed the gallic acid interacting with the key amino acid residues: TRP87 of NS5 MTase (Figure 4a) in addition to GLN760, SER776, CYS780, and ASP808 of NS5 RdRp (Figure 4b) through hydrogen bonds.

### 2.4. The Antiviral Effects of T. bellirica Extract and Triphala F3 against DENV Infection in Hepatocellular Huh7 Cells

**Figure 5:**
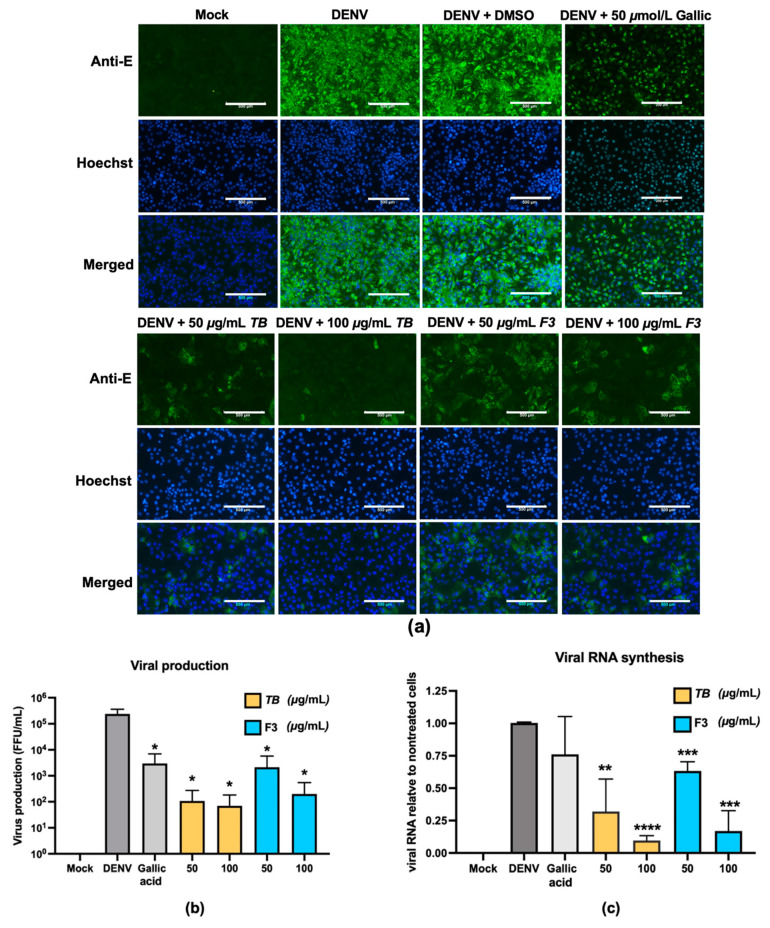
Antiviral activity of gallic acid, *T. bellirica* (TB) extract and Triphala formula 3 (F3) on inhibiting virus infectivity in Huh7 hepatoma cells (**a**) The effects of gallic acid, TB and F3 in lowering the viral infectivity was accessed by IFA. (**b**,**c**) Treatment of gallic acid, TB and F3 decreased the level of viral production and viral RNA synthesis, judged by viral titration and real-time PCR, respectively. The statistical difference was tested by using Student’s *t*-test which * indicates *p* < 0.05; ** indicates *p* < 0.01; *** indicates *p* < 0.001 and **** indicates *p* < 0.0001.

To evaluate the effects of *T. bellirica* extract and Triphala F3 in human cells, Huh7 hepatocellular carcinoma was selected based on the evidence that liver is a critical target organ of DENV. Huh7 cells were infected with DENV and treated with 50 μmol/L of gallic acid or 50 and 100 μg/mL of *T. bellirica* extract and Triphala F3. Then the effects—lower infected cell numbers, viral production, and genomic RNA replication—were investigated. The immunofluorescence assay demonstrated a dramatic reduction of infected cells in *T. bellirica* extract or Triphala F3-treated cells (Figure 5a). At an equal concentration, *T. bellirica* extract treatment was shown to lower the number of infected cells to a greater extent than observed in Triphala F3. We further investigated its effects on inhibiting the production of new viruses in the culture’s supernatant. The result showed that treatment with gallic acid, *T. bellirica* extract, and Triphala F3 significantly lowered the virus titer in a dose-dependent manner (Figure 5b). Treatment with 50 μmol/L of gallic acid, 100 μg/mL of *T. bellirica* extract, and Triphala F3, caused a reduction of the titer to 3.0 × 10^3^, 7.0 × 10^1^, and 2.0 × 10^2^ FFU/mL, respectively compared to 2.4 × 10^5^ FFU/mL in the nontreated control (Figure 5b).

Based on the molecular docking results, which showed gallic acid could bind to NS5 protein, we thus hypothesized that they might affect the level of viral genomic RNA. A real-time PCR was performed to determine the level of genomic RNA in infected cells after treatment of gallic acid, *T. bellirica* extract and Triphala F3. These results revealed, concordantly with the immunofluorescence assay and viral titration, that the viral RNA was significantly decreased in gallic acid-, *T. bellirica* extract-, and Triphala F3-treated cells (Figure 5c). The gallic acid, at the concentration of 50 μmol/L, caused the reduction of genomic RNA to 0.76 relative to that of the non-treatment control, which was set as 1.0 (Figure 5c). In addition, the treatment with 50 and 100 μg/mL *T. bellirica* extract reduced the viral genomic RNA to 0.32 and 0.09, respectively, relative to that of the non-treatment control, whereas treatment of 50 and 100 μg/mL Triphala F3 lowered the viral RNA to 0.63 and 0.17, respectively (Figure 5c).

**Figure 6:**
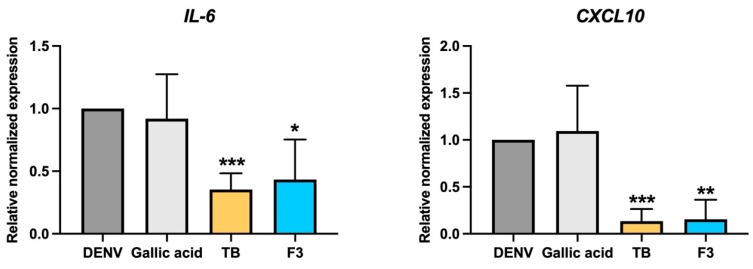
The effect of gallic acid, *T. bellirica* extract and Triphala F3 in decreasing DENV-2 induced IL-6 and CXCL-10 expression. DENV-2-infected Huh7 cells were treated with 50 μmol/L gallic acid, 100 μg/mL of *T. bellirica* extract, and Triphala. The gene expression levels of *IL-6* and *CXCL-10* were evaluated and normalized with *GAPDH*, at 24 h after infection, by using real-time PCR and represented as the values relative to the normalized expression. The statistical difference was tested by using Student’s *t*-test which * indicates *p* < 0.05; ** indicates *p* < 0.01; *** indicates *p* < 0.001.

The effects of gallic acid, *T. bellirica* extract and Triphala F3 treatment in diminishing the levels of cytokine expression were further investigated. The upregulation of IL-6 and CXCL-10 expression had been reported previously as being associated with liver dysfunction in dengue patients [11]. Decreasing IL-6 and CXCL-10 expression might theoretically lower the risk of liver pathogenicity caused by DENV infection. We determined the mRNA expression level of *IL-6* and *CXCL-10* after 24-h treatment with 50 μmol/L of gallic acid or 100 μg/mL of *T. bellirica* extract and Triphala F3 in DENV infected Huh7 (Figure 6). The results showed significantly lower expression of *IL-6* to 0.35 and 0.43 folds in *T. bellirica* extract and Triphala F3 treated cells, respectively, relative to the non-treatment control (set as 1.0). Moreover, the treatment caused the significant reduction of *CXCL-10* expression to 0.13 and 0.15 folds in *T. bellirica* extract and Triphala F3 treated cells, respectively. However, no significant difference was observed in the gallic acid-treated cells.

## 3. Discussion

DENV infection is a global public health problem. The number of infected cases has been continuously increasing annually, reflecting the fact that public policies to control the spread of the disease and lower the risk of severe cases has been inadequate. As a result, DENV is now endemic in more than 100 countries of Africa, the Americas, the eastern Mediterranean, south-east Asia, and the western Pacific [7]. Accordingly, the development of anti-DENV agents is needed to control the serious situation. Medicinal plants are attractive sources for antiviral agent investigation, based on their diverse biological properties; in addition, the traditional use of these medicinal plants clearly demonstrates their safety for human use. However, scientific validation is necessary to approve the therapeutic use of medicinal plants. Herein, we have demonstrated the effect of traditional Ayurvedic medicine Triphala and its individual extracts in inhibiting DENV infection in vitro.

Triphala, widely used in Indian traditional medicine, has potential for several clinical uses, based on its anti-bacterial, anti-fungal, anti-allergic, anti-inflammatory, and antioxidative properties [12,13]. Triphala has been recommended for treating various ailments such as being a cardiotonic, controlling blood pressure, improving blood circulation, and reducing cholesterol levels, as well as immunomodulatory properties to improve the body’s defense systems [1,12,13]. However, its antiviral activity against DENV infection has not been reported, yet. In the present study, the inhibitory effect was firstly tested in Vero cells, which revealed the high efficiency of *T. bellirica* extract and Triphala F3 in lowering the intracellular E antigen and the number of infected cells (Figure 1). Interestingly, Triphala F3 corresponded to the combination of *P. emblica, T. chebula*, and *T. bellirica* extracts at a 3:1:2 ratio. This formulation had more potential than Triphala F1, in which *T. bellirica* extract was present in the highest proportion, with an extract ratio of 1:2:3. The HPLC profile demonstrated that gallic acid, as the major compound of these extracts (Figure 2) and the antiviral assay revealed the ability of gallic acid to inhibit DENV infection in a dose-dependent manner (Figure 3) which emphasizes the anti-DENV contribution of Triphala and its individual constituents. Although the gallic acid could promote the antiviral activity on DENV infection, we could not, however, exclude the contribution of the other substances in the extracts, especially those which were shared among the three extracts. To find out the candidate bioactive compounds in the extracts, we performed GC–MS/MS to identify the compounds shared among the extracts. The GC–MS/MS analysis showed that there were a total of nine compounds shared between the three extracts including gallic acid, quercetin, 3-furaldehyde, 4-cyclopentene-1,3-dione, 5-methylfurfural, 3-ethyl-3-methylheptane, 3-furanmethanol, 1,2-cyclopentanedione, 2,4-di-tert-butylphenol (Table S1). However, due to the difference of methodology, we could not compare the identified compounds to the HPLC profile. Therefore, the phytochemical validation of these compounds, as well as their bioactivity to inhibit DENV infection, are in need of further investigation.

The variation of addition time suggested the gallic acid disrupted the DENV infectivity both before and after the virus entry steps, a behavior which is similar to a previous report which showed the inhibitory effects of gallic acid in both pre-treatment and post-treatment conditions [14]. Trujillo-Correa et al. reported the bark extract of *Psidium guajava *L. which contained gallic acid, quercetin and catechin as the bioactive compounds exerted the high potency to inhibit DENV in Vero cells [14]. Consistent with their results, our research showed that the treatment of gallic acid after the virus entry was more effective over the treatment during or prior to the virus entry (Figure 3), suggesting the potential target of gallic acid could exist inside the cells.

Indeed, the interruption of any step during the life cycle of DENV infection could result in the inhibition of the infection. During the infection, DENV proceeds through multiple steps to finish replication and produce new progeny from the infected cells, namely: virus entry (receptor binding, endocytosis), fusion, uncoating, RNA replication, translation, protein processing/cleavage, assembly, maturation, and exocytosis [15]. It is not only the viral proteins that function through the life cycle; the host proteins also participate in several critical steps that directly affect the virus’ multiplicity, in addition to modulating the host response [15]. Thus, it is possible that the antiviral activity might be the consequences of the interaction of a bioactive compound with virus protein and/or host protein. In our study, the prediction of gallic acid and NS5 interaction was performed to demonstrate the possibility that gallic acid inhibited DENV infection via targeting the virus’ proteins. The docking scores achieved from molecular docking suggested that the gallic acid was favorable to bind to two domains of the NS5 protein, including MTase and RdRp (Figure 4), which, in accordance with the time of addition assay, shows that the most effective mode of action of gallic acid was observed post-infection, wherein the gallic acid was added after the virus entry step. NS5 protein is a bi-functional protein (MTase and RdRp) necessary for the replication steps of DENV genomic RNA. The inhibition of NS5 significantly affected the DENV life cycle, providing evidence for NS5 as one of the main targets of an anti-DENV inhibitor [16,17,18]. Interestingly, gallic acid has been reported to interact with the NS5 and E proteins of DENV [14], in accordance with our study. This result emphasized the strong possibility that gallic acid may interact with the NS5 protein of DENV to inhibit its RNA synthesis, which eventually results in the inhibition of infection. However, we did not exclude the E protein from the action of gallic acid, since it might partially contribute to its anti-viral activity, as pre-treatment slightly inhibited viral infection (Figure 3a).

On the other hand, since gallic acid is well-known for its antioxidative activity, another mechanism of its antiviral activity might be through modulating the host response to fighting the viral infection, particularly the antiviral response. Olagnier and colleagues reported on the host response to DENV infection in monocyte-derived dendritic cells (Mo-DC) by using a genome-wide transcriptome analysis that demonstrated that the major responses are promoted via the activation of the IRF3/7/STAT1 and NF-κB-driven antiviral and inflammatory networks, as well as the stimulation of an oxidative stress response [19]. Controlling the oxidative stress response was critical for the induction of antiviral, inflammatory, and cell death responses and DENV infection stimulated the nuclear factor erythroid 2-related factor 2 (Nrf2) -dependent antioxidant gene transcriptional program. Nrf2 has been documented as the major contribution to cellular defense against oxidative damage. Nrf2 regulated DENV infection and modulated the innate immune and apoptotic responses, whereas downregulation of Nrf2 by RNA interference has been shown to increase immune and apoptotic responses in Mo-DC [19]. These results revealed the critical role of Nrf2 during DENV infection and that the manipulation of its levels might be beneficial in controlling DENV replication. Interestingly, gallic acid has been reported, previously, to promote the Nrf2-antioxidative response element signaling pathway in a model of inflammation and oxidative stress induced by particulate matter (PM10) [20]. Importantly, treatment with gallic acid not only restored Nrf2 expression in PM10-induced A549 cells and lung tissue from mice models, but caused the reduction of proinflammatory genes, IL6, which were similarly found in our results after treatment of the infected cells with the extracts (Figure 6). Since gallic acid was able to modulate the host response, we could not exclude this effect, which might partially account for its anti-viral activity. However, further investigation to confirm the involvement of gallic acid in modulating the Nrf2 mechanism in the DENV infection model is required.

The liver is a critical target organ of DENV infection, as evidenced by several pathogenesis studies showing that virus replications were frequently detected in the autopsy liver samples of fetal cases [21]. Hepatic histological changes have been reported, including moderate midzonal hepatocyte necrosis, microvascular steatosis, and councilman bodies [22]. Furthermore, abnormal elevated liver enzyme levels in patients with severe diseases are commonly higher than uncomplicated dengue fever [23,24] suggesting liver injury participates in DENV pathogenesis. The present study demonstrated that treatment with *T. bellirica* extract or Triphala could significantly lower the infection of DENV (Figure 5). The infection in Huh7 caused over 2.4 × 10^6^ FFU/mL at 48 h of infection, whereas the treatment of *T. bellirica* extract or Triphala was able to dramatically diminish the virus titer approximately 10^4^- and 10^3^-fold, respectively. Higher viral titers may cause greater consequence in such infection; thus, the lower viral titer observed from *T. bellirica* extract- or Triphala-treated cells would lower the risk of liver pathogenesis as well as disease severity related to liver function.

The involvement of immunopathogenic mechanisms on liver injury, hypothesized according to immunohistochemical study, revealed the infiltration of inflammatory cells in the liver biopsies of patients with high level of aminotransferases (AST) [22]. Cytokine storm is a unique characteristic of DENV infection, and high levels of circulated cytokines were reported to be associated with DENV severity. However, only a few cytokines/chemokines have been reported to participate in liver immunopathogenicity. The DENV infection of primary hepatocytes, ex vivo, revealed an increase in the expression levels of IL-6 and IL-8, which are involved in acute inflammatory responses [25]. Moreover, it promoted immune-attractive chemokines, such as Interferon gamma-induced protein 10 (IP-10/CXCL10). Interestingly, treatment with *T. bellirica* extract or Triphala upon DENV infection could significantly lower the expression levels of IL-6 and IP-10 in Huh7 cells (Figure 6). The significance of high-level IL-6 in the plasma of DENV-infected persons has been well documented [26,27]. High levels of IL6 might be involved in severe dengue pathogenesis due to its correlation with disease severity [27]. The role of CXCL10/IP10 has been demonstrated in DENV severity associated with the induction of vascular leakage and a novel association with changes in liver dysfunction [11]. The level of circulated IP-10 was positively related with dengue hemorrhagic fever and pulmonary effusion or ascites [11]. Interestingly, the increase in IP-10 circulation correlated to abnormal liver function (increases in AST levels with *p* < 0.0001). In addition, the presentation of hepatomegaly appeared higher in DENV patients with high levels of IP-10, compared with patients without physical liver alteration [11]. The accumulative evidence suggests that the increase of severity-related cytokine/chemokine levels was critical in DENV treatment and treatment with *T. bellirica* extract or Triphala, which resulted in lowering IL-6 and IP-10, and would provide clinical benefit by limiting the severity of the disease.

Traditional use of Triphala as a therapeutic option or to promote immunity function in healthy people, suggests a high level of safety for human use. Clinical trials of Triphala in healthy Thai donors orally administered Triphala with meals, three times daily (1050 mg/d) for two weeks, demonstrated no adverse effects [28]. On the contrary, such consumption showed immunomodulation activity, significantly increasing cytotoxic T cells (CD3−CD8+) and natural killer cells (CD16+CD56+) [28]. Interestingly, these immune cells also play important roles in combatting infection by the virus as the defensive mechanism of immunity, which might be beneficial in DENV treatment. The current study showed the efficiency of *T. bellirica* extract or Triphala over gallic acid alone, theoretically due to the extracts’ ability to provide synergistic effects from numerous bioactive substances. However, additional investigation and clinical development is required to support the traditional therapeutic use of *T. bellirica* extract or Triphala in DENV treatment. This should provide not only an alternative approach for DENV treatment but also encourage traditional medicinal use globally, while increasing demand and promoting traditional medicine conservation which would greatly contribute to the world sustainable development goal.

## 4. Materials and Methods

### 4.1. Cell Culture and Virus Propagation

Vero cells (kidney epithelial cells isolated from African green monkeys) were obtained from American Type Culture Collection (ATCC) (Manassas, VA, USA) and grown in minimal essential medium (MEM) supplemented with 10% (*v*/*v*) fetal bovine serum (FBS) and 2 mM glutamine (Gibco, Thermo Fisher Scientific, Waltham, MA, USA). Huh7 cells (hepatocellular carcinoma cell line) were obtained from the Japanese Collection of Research Bioresources Cell Bank (JCRB0403) (Osaka, Japan) and grown in Dulbecco’s Modified Eagle Medium/Nutrient Mixture F-12 (DMEM/F-12) supplemented with 10% (*v*/*v*) FBS and 2 mM glutamine (Gibco, Thermo Fisher Scientific, Waltham, MA, USA) at 37 °C with 5% CO_2_. The propagation of DENV serotype 2 (DENV2) strain Thailand/16681/84 [NCBI: txid31634] was performed in C6/36 cells using a multiplicity of infection (MOI) of 0.1 at 28 °C in Leibowitz-15 culture medium (Gibco, Thermo Fisher Scientific, Waltham, MA, USA) supplemented with 1% FBS and 10% tryptose phosphate broth (TPB). The culture supernatant was harvested at day 5 after infection and stored at −70 °C until use.

### 4.2. Plant Materials

Fresh fruits of Indian gooseberry (*Phyllanthus embrica *L.) or (*Emblica officinalis Geartn*.), *Terminalia bellirica* (Gaertn.) Roxb., and *Terminalia chebula* Retz. were purchased from Kad Khongkong organic market organized by the Faculty of Agriculture, Chiang Mai University, Muang Chiang Mai district, Chiang Mai, Thailand during September–October 2019 (Figure S2, Supplementary Materials). All specimens were identified by Dr. Narin Printarakul, taxonomist, Department of Biology, Faculty of Science, Chiang Mai University. The voucher specimens’ number PE001CM, TB001CM and TC001CM of each plant species were kept in CMUB herbarium (Department of Biology, Faculty of Science, Chiang Mai University).

### 4.3. Plant Extraction

The fresh fruits were washed and dried in a hot air oven at 60 °C for 24 h. The dried samples (1 kg) were ground using electronic blender into powder followed by maceration with 70% ethanol at 1:20 *v*/*v*. The extract was shaken at 160 rpm at room temperature for 12 h. After maceration, each extract solution was filtered through Whatman^®^ filter paper no. 1 using vacuum suction. The marc was exhaustively macerated for other twice times. All filtrates were pooled together and concentrated using a rotary evaporator and poured into brown glass bottles to dry on 95 °C water bath. The extracts were kept in brown bottles at 4 °C until use.

### 4.4. Preparation of Triphala Formula

The stock solution of extracts was prepared in DMSO at the concentration of 125 mg/mL. The extracts were formulated by mixing into three formulations with different ratios according to traditional Thai herbal medicine. Triphala F1 was the combination of *P. emblica, T. chebula*, and *T. bellirica* extracts at a ratio of 1:2:3, Triphala F2 was 2:3:1, and Triphala F3 was 3:1:2, respectively.

### 4.5. High Performance Liquid Chromatography (HPLC)

The gallic acid phytochemical compound composition in each plant extract was determined by high performance liquid chromatography (HPLC). The compounds of gallic acid (Sigma-Aldich, Darmstadt, Germany) in plant extracts were optimized for detection by gradient HPLC systems with a ZORBAX Eclipse XDB-C18 column (4.6 × 150 mm, 5429 μm; Agilent Technologies, Santa Clara, CA, USA) at ambient temperature. The mobile phase consisted of water with 0.5% glacial acetic acid (solvent A) and methanol (solvent B). The gradient steps were adjusted with modifications: 100% A, 0–20 min; 50% A, 20–30 min; 40% A, 30–35 min; 30% A, 35–40 min; 20% A, 40 min; post-time, 5 min before next injection. The flow rate was 1.0 mL/min, and the injection volume was 20 μL. A UV photodiode array detector (270 nm) was used to monitor the wavelength.

### 4.6. Validation of HPLC

Authenticated gallic acid compound and three Triphala formulations were dissolved in methanol (HPLC grade). The method was validated according to ICH guideline for linearity, precision, accuracy, limit of detection, and limit of quantification. The linearity of the method was determined at six concentration levels of each standard, ranging from 12.5 to 500 μg/mL. The standard was run in triplicate and assessed by means of linear regression. The limit of detection (LOD) and the limit of quantification (LOQ) of the developed method were determined by injecting progressively lowed concentrations of the standard solutions. The LOD and LOQ of marker compounds were calculated at a signal-to-noise ratio of approximate 3:1 and 10:1, respectively. The accuracy of the method was determined by recovery experiments. The Triphala F1 formulation (100 μg/mL) was spiked with three different amounts of standard compounds. The spiked samples were analyzed by the described method. Then, percentage recovery and the relative standard deviation of recovery was calculated. The precision of the method was demonstrated by intra-day and inter-day variation studies. Six replicate injections of standards were injected, and percent relative standard deviations (%RSD) were calculated.

### 4.7. Folin–Ciocalteu Method

The total phenolic content was determined using the Folin–Ciocalteu method. The extracts and Triphala formulations were prepared and mixed with 50% Folin–Ciocalteu’s reagent (Merck, Kenilworth, NJ, USA). Then the mixture was incubated in the dark for 5 min. After that, 5% sodium carbonate was added, followed by incubation for 1 h. The reaction was measured at an absorbance frequency of 725 nm. The total phenolic content was expressed as mg of gallic acid equivalents per gram extract (mg GAE/g extracts).

### 4.8. DPPH (2,2-Diphenyl-1-picryl-hydrazyl-hydrate) Free Radical Method

The antioxidative activity of each extract and Triphala formulation was determined by 2,2-diphenyl-1-picrylhydrazyl (DPPH) assay. The concentration of extracts and Triphala formulation were prepared with a range of 10–60 µg/mL of methanol. Then, the solution was mixed with 0.1 mM DPPH reagent (Sigma-Aldrich, Darmstadt, Germany) and incubated for 20 min at room temperature in the dark. The absorbance was measured at a wavelength of 517 nm. The scavenging activity was evaluated and compared to the gallic acid standard curve. The antioxidative activity was expressed as mg of gallic acid equivalents per gram extract (mg GAE/g extracts).

### 4.9. Virus Infection and Gallic Acid/Herb Extracts/Triphala Formulation Treatment

For the virus infection, Vero cells were seeded at 2 × 10^4^ cells per well in a 96-well plate for 24 h before the experiments. DENV2 of approximately 4 × 10^3^ FFU/mL FFU was prepared in 2% FBS containing MEM media and added to the Vero cells (100 μL per well) and further incubated for 2 h at 37 °C with 5% CO_2_ to allow absorption of DENV into the cells. The cells were then washed twice and replaced with the 100 μL fresh media without or with gallic acid (Sigma-Aldrich Corporation, St. Louis, MO, USA), herb extracts, or Triphala formulation at the indicated concentrations. The cells with no infection, called the mock control, was set as the negative control for the experiment. The infected cells and culture supernatant were collected at 48 h after infection for the cell-based ELISA.

### 4.10. Cell-Based ELISA

Cell-based ELISA was carried out to determine the inhibitory effect. After 48 h of virus infection without or with gallic acid (Sigma-Aldrich Corporation, St. Louis, MO, USA), herb extracts, or Triphala formulation treatment, the infected cells were harvested and stained for intra-cellular viral protein antigen. The infected cells were fixed with 4% paraformaldehyde at room temperature for 15 min and washed twice with ice-cold phosphate-buffered saline (PBS) before permeabilized with 0.2% Triton X-100 (15 min at room temperature). The cells were blocked with 1% bovine serum albumin (BSA) in PBS for 30 min and incubated for at least 3 h with monoclonal anti-DENV E antibody clone 4G2 at 37 °C. Cells were washed three times with PBS containing 0.5% Tween-20 (PBST) and further incubated for 30 min followed by horseradish peroxidase-conjugated rabbit anti-mouse IgG (Dako, CA, USA) (at 1:2000 dilution). Cell plates were washed before adding TMB (3,3,5,5-tetramethylbenzidine) substrate (Invitrogen, Carlsbad, CA, USA), and the absorbance at an optical density (OD) of 650 was measured, wherein the mock control was set as blank. The absorbance was used to calculate the % E antigen compared to that of the non-treatment control (set as 100% E antigen) as the following equation:

% E antigen = (absorbance of tested condition/absorbance of non-treatment control) × 100

### 4.11. Immunofluorescence Assay (IFA)

Immunofluorescence assay (IFA) was performed to measure the reduction in the infection rate. Vero cells or Huh7 cells were seeded at 1 × 10^5^ cells per well in a 24-well plate for 24 h before the experiments. DENV2 of approximately 4 × 10^4^ FFU/mL was prepared in 2% FBS containing culture media and added to Vero cells or Huh7 cells (400 μL per well), added to the cells, and further incubated for 2 h at 37 °C with 5% CO_2_, to allow the absorption of DENV into the cells. The unbound viruses were removed and washed the fresh media before adding fresh media, without or with gallic acid (Sigma-Aldrich Corporation, St. Louis, MO, USA), herb extracts, or Triphala formulation at the indicated concentrations (400 μL per well). Forty-eight hours after infection, the infected cells were harvested and fixed with 4% paraformaldehyde at room temperature for 15 min and washed twice with ice-cold phosphate-buffered saline (PBS) before permeabilized with 0.2% Triton X- 100 (15 min at room temperature). The cells were blocked with 1% bovine serum albumin (BSA) in PBS for 30 min and incubated at least for 3 h with 4G2 monoclonal anti-DENV E antibody (300 μL/well) at 37 °C. Cells were washed three times with PBS containing 0.5% Tween-20 (PBST) and further incubated for 30 min, followed by Alexa Fluor 488 goat anti-mouse IgG (dilution 1:1000) (Invitrogen, Carlsbad, CA, USA). The nuclei were stained with Hoechst^®^ 33342 nucleic acid stain (dilution 1:1000) (Invitrogen, Carlsbad, CA, USA). The stained cells were washed three times with 0.5% PBST and monitored under a fluorescence microscope (Nikon Instruments, Inc., Melville, NY, USA).

### 4.12. Virus Titration and FFU Staining

The culture supernatant of Huh7 was harvested after 48 h of infection without or with treatment by gallic acid (Sigma-Aldrich Corporation, St. Louis, MO, USA), herb extracts, or Triphala formulation, as per the methods described above. Virus titers in the culture supernatants were determined by FFU staining. In brief, the Vero cells were plated at 2 × 10^4^ cells per well in a 96-well plate. The DENV was prepared by 10-fold serial dilution in 2% FBS containing MEM media and added to the cells (100 μL per well). The virus was allowed to infect the cells for 2 h at 37 °C with 5% CO_2_, to allow absorption of DENV into the cells. The unbound viruses were removed and overlaid with 2% FBS MEM media containing 2% carboxymethylcellulose. The plate was then further incubated for 72 h at 37 °C with 5% CO_2_. Foci formation assay was used to evaluate DENV-infected cells. The infected cells were fixed with 4% paraformaldehyde at room temperature for 15 min and washed twice with ice-cold phosphate-buffered saline (PBS) before permeabilized with 0.2% Triton X- 100 (15 min at room temperature. The cells were blocked with 1% bovine serum albumin (BSA) in PBS for 30 min and incubated at least for 3 h with 4G2 monoclonal anti-DENV E antibody at 37 °C. Cells were washed three times with PBS containing 0.5% Tween-20 (PBST) and further incubated for 30 min, followed by horseradish peroxidase-conjugated rabbit anti-mouse IgG (Dako, Santa Clara, CA, USA) (at 1:2000 dilution). Clusters of infected cells were detected using a 3,30-diaminobenzidine (DAB) substrate (Sigma-Aldrich Corporation, St. Louis, MO, USA). The DENV foci were counted manually under a light microscope (20 × magnification) (Nikon Instruments, Inc., Melville, NY, USA) and FFU/mL was calculated.

### 4.13. Real-Time PCR (qRT-PCR)

In brief, Huh7 cells were seeded at 1.5 × 10^5^ cells per well in a 12-well plate for 24 h before the experiments. DENV2, of approximately 4 × 10^4^ FFU/mL, was added to the cells and further incubated for 2 h at 37 °C with 5% CO_2_ to allow absorption of DENV into the cells and replaced with fresh media without or with gallic acid (Sigma-Aldrich Corporation, St. Louis, MO, USA), herb extracts, or Triphala formulation at the indicated concentrations. Twenty-four hours after infection, the infected cells were collected and isolated for total RNA by using TRIzol reagent (Invitrogen, Carlsbad, CA, USA). Approximately, 500 ng of total RNA was used in the template for cDNA synthesis by using a cDNA synthesis kit (Meridian Bioscience, Cincinnati, OH, USA). The 0.5 μL of cDNA was a template for the real-time PCR reaction using 2 × SensiFAST SYBR No-ROX Mix (Meridian Bioscience, Cincinnati, OH, USA and the primers specific to DENV genome (Forward 5′ CCGGCTCTACTCCTATGATG 3′; Reverse 5′ ATCCAGATGTCATCAGGAAAC 3′); IL-6 (Forward 5′ GTACATCCTCGACGGCATC 3′; Reverse 5′AGCCACTGGTTCTGTGCCT 3′); CXCL-10 (Forward 5′GGATCGGCCATCAAGAA 3′; Reverse 5′AAGCAGGGTCAGAACATCCA 3′); and GAPDH (Forward 5′CGACCACTTTGTCAAGCTCA 3′; Reverse 5′AGGGGTCTACATGGCAACTG 3′). The PCR reactions were conducted as per the following profile: pre-denaturation: 95 °C, 30 s followed by denaturation: 95 °C, 10 s, annealing: 59 °C, 10 s and extension: 72 °C, 15 s for 40 cycles. The data was collected and analyzed for cycle threshold by using the default settings of an iCycler Thermal Cycler (Bio-Rad Laboratories, Hercules, CA, USA). The cycle threshold values were normalized with house-keeping genes (GAPDH) with the 2^−ΔΔCT^ method and compared to a relative value of 1.0, representing the non-treatment control. The data are represented in relative normalized expression values.

### 4.14. Molecular Docking

The three-dimensional structures of the DENV NS5 proteins, including MTase (PDB entry 1r6A) and RdRp (PDb entry 5JJR), were extracted from the Protein Data Bank (https://www.rcsb.org/ accessed on 19 November 2021). The protein receptor was prepared by removing water and other atoms, and then by adding a polar hydrogen group, using Biovia Discovery Studio Client 2020 (Dassault Systèmes BIOVIA, Discovery Studio Modeling Environment, Release 2017, San Diego: Dassault Systèmes, 2016). The ligand structures of gallic acids was derived from MolView (https://molview.org/ accessed on 19 November 2021) and the forcefield for molecular docking was optimized by using a UCSF Chimera program [29]. The molecular docking of the protein receptors and gallic acid was performed by using the SwissDock web-based server (http://www.swissdock.ch/ accessed on 19 November 2021). The docking pose analysis was performed by UCSF Chimera program. The interaction of receptor and ligand was analyzed by using the “view interaction” mode of Biovia Discovery studio client 2020 to present the interaction in 2D diagrams.

### 4.15. Statistical Analysis

Mean and standard deviation (SD) were analyzed from a minimum of three independent experiments. Statistical analyses were performed using GraphPad Prism Software version 8 (GraphPad Software, Inc., La Jolla, CA, USA). Results were analyzed for statistical differences using Student’s *t*-test (* indicates *p* < 0.05; ** indicates *p* < 0.01; and, *** indicates *p* < 0.001).

## 5. Conclusions

DENV infection emerges as a global public health problem which more than 2.5 billion people are currently at risk. Accordingly, the development of anti-DENV agents is needed to control the spread of the disease and lower the risk of severe cases. Herein, we demonstrated the efficiency of the most well-known herbal formulation in ayurvedic medicine Triphala and its individual extract constituents to inhibit DENV-2 infection. Treatment with *T. bellirica* extract or Triphala formulated with a high ratio of *T. bellirica* extract called Triphala F3 revealed the remarkable efficiency in significantly lowering DENV infection judged by ELISA and IFA. The antiviral effect to inhibit the virus production and RNA replication emphasizing the potential of *T. bellirica* extract or Triphala F3 on DENV infection inhibition. The HPLC analysis demonstrated the gallic acid as the bioactive compound which be confirmed for its ability to lower the virus infection at post entry step. The molecular docking pointed that gallic acid might interact to DENV NS5 protein which could promote the inhibition of viral RNA replication resulting in the infection inhibition. Taken together, the finding of this study demonstrated the potential of Triphala and *T. bellirica* extract as a therapeutic option for DENV infection treatment.

## Data Availability

Data is contained within the article or supplementary material.

## Supplementary Materials

The following are available online at https://www.mdpi.com/article/10.3390/ph14121236/s1, Figure S1: Effect of *P. emblica*, *T. chebula*, and *T. bellirica* extracts and Triphala formulation treatment on Vero cell viability, Table S1: The chemical compositions of *P. emblica*, *T. chebula*, and *T. bellirica* ethanolic extract by GC-MS analysis.
