# Supplementary material for: Triphala in Traditional Ayurvedic Medicine Inhibits Dengue Virus Infection in Huh7 Hepatoma Cells
Source: Pharmaceuticals (Basel). 2021 Nov 28;14(12):1236. doi: 10.3390/ph14121236 (PMC8708800; doi:10.3390/ph14121236)
Supplement: Supplementary file 1 [file pharmaceuticals-14-01236-s001.zip › pharmaceuticals-1447225-supplementary.pdf]

Supplementary Materials

Triphala in traditional Ayurvedic medicine inhibits dengue virus infection in Huh7 hepatoma cells

Aussara Panya1,2, *, Kanyaluck Jantakee1, Suthida Punwong1, Supawadee Thongyim1, Thida Kaewkod1, Pa-thai Yenchitsomanus3, Yingmanee Tragoolpua1,2, Hataichanok Pandith1,2, *

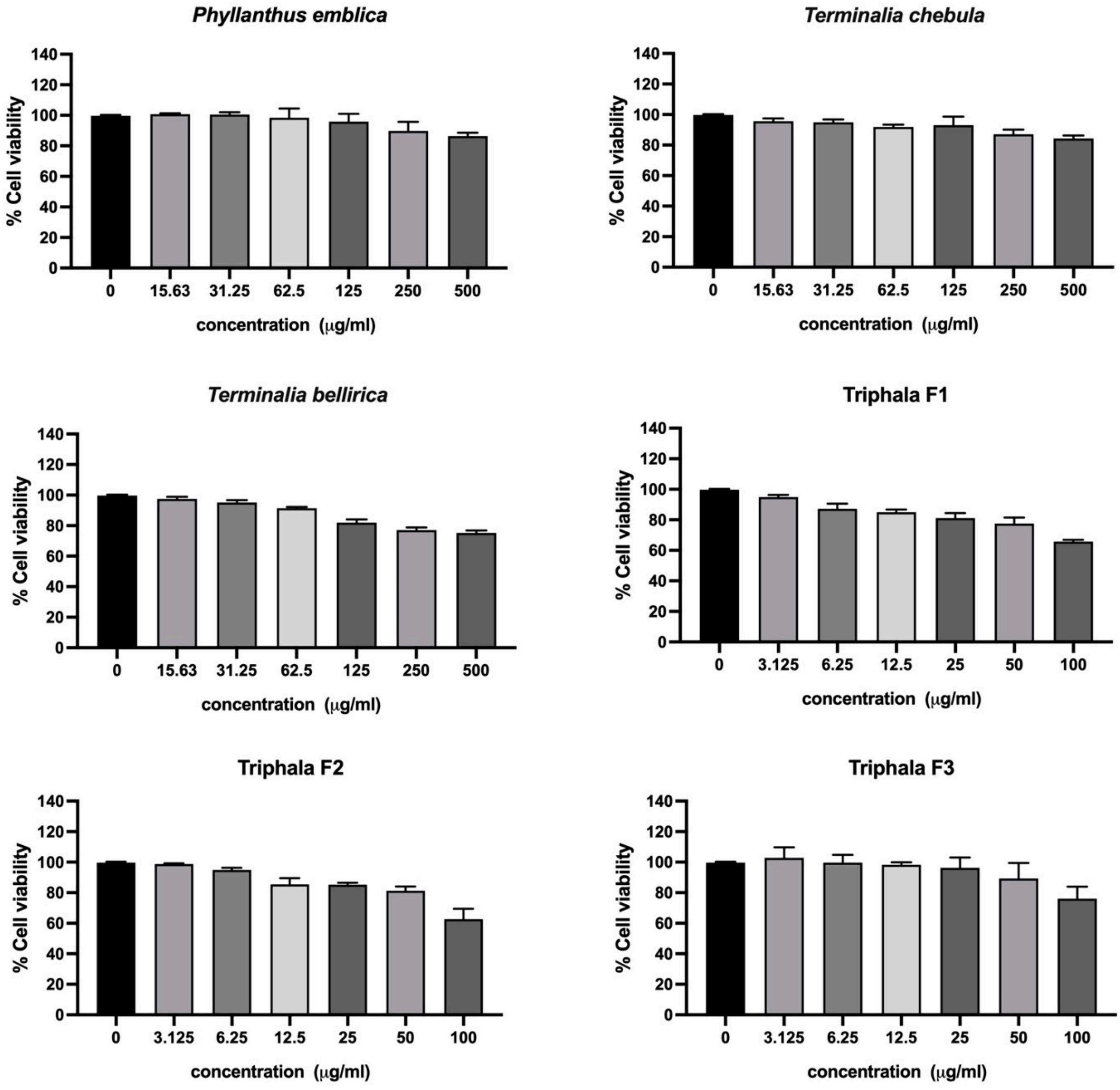

Figure S1: Effect of *P. emblica*, *T. chebula*, and *T. bellirica* extracts and Triphala formulation treatment on Vero cell viability. The toxicity of *P. emblica, T. chebula*, and *T. bellirica* extracts and three formulations of Triphala was determined by using the cell viability assay. The extracts were prepared in different concentrations ranging from 0-100 µg/mL in culture media and added to the Vero cells (100 µL per well). The cell viability was measured at 48 hours after treatment using the PrestoBlue™ reagent which monitored the reducing capacity of the living cells. The absorbance was measured at OD 570 where OD595 was used as the reference OD. The changes of absorbances were used to calculate the percentage of cell viability relative to non-treatment control (set as 100%). The graph represents the percentage of cell viability in mean ± SD.

Table S1: The chemical compositions of *P. emblica, T. chebula, and T. bellirica* ethanolic extract by GC-MS analysis. The phytochemical investigation of Triphala leaf extract was performed by using GC-MS equipment (GC 7890B Agilent Technology) with a DB-5MS Agilent fused silica capillary column (30 x 0.25 mm ID; film thickness: 0.25 μm). Experimental conditions of the GC-MS system were as follows: flow rate of mobile phase (carrier gas: He) was set at 1.0 mL/min. Samples dissolved in ethanol and the injection quantity of 1 μL was used (split ratio 10:1). The column oven temperature was set at 50 °C for 2 min, raised at 5 °C/per min up to 230 °C for 1 min, 5°C/per min up to 280°C for 2 min, and the final temperature was increased to 300 °C for 5 min. The phytochemicals were identified based on a comparison of their retention time (min), peak area, and mass spectral patterns with that spectral database of authentic compounds stored in the National Institute of Standards and Technology (NIST17) library.

No.	Classification	IUPAC Name	Name of compounds	Molecular Formula	RT
1	Hydrocarbons	furan-3-carbaldehyde	3-Furaldehyde	$C_5H_4O_2$	6.5
2		cyclopent-4-ene-1,3-dione	4-Cyclopentene-1,3-dione	$C_5H_4O_2$	7.7
3		5-methylfuran-2-carbaldehyde	5-Methylfurfural	$C_6H_6O_2$	10.0
4		3-ethyl-3-methylheptane	3-Ethyl-3-methylheptane	$C_{10}H_{22}$	14.2
5	Carboxylic Acids	furan-3-ylmethanol	3-Furanmethanol	$C_5H_6O_2$	7.0
6		cyclopentane-1,2-dione	1,2-Cyclopentanedione	$C_5H_6O_2$	8.9
7		3,4,5-trihydroxybenzoic acid	Gallic acid	$C_7H_6O_5$	10.9
8	Flavonoids	2-(3,4-dihydroxyphenyl)-5,7-dihydroxy-3-[(2R,3R,4S,5R,6R) 3,4,5-trihydroxy-6-(hydroxymethyl)oxan-2-yl]oxychromen-4-one	Quercetin	$C_{15}H_{10}O_7$	22.8
9	Phenols	2,4-ditert-butylphenol	2,4-Di-tert-butylphenol	$C_{14}H_{22}O$	25.5
